# Biocontrol Efficiency of *Leuconostoc mesenteroides* GY-2 Against Postharvest Black Rot Caused by *Alternaria alternata* and the Mechanisms of Action

**DOI:** 10.3390/jof11100705

**Published:** 2025-09-29

**Authors:** Pengbo Dai, Bing Li, Yanan Li, Li Wang, Tongle Hu, Yanan Wang, Xianglong Meng, Bo Li, Keqiang Cao, Shutong Wang, Manli Sun

**Affiliations:** State Key Laboratory of North China Crop Improvement and Regulation/College of Plant Protection, Hebei Agricultural University, Baoding 071001, China; daipengbo@hebau.edu.cn (P.D.);

**Keywords:** biological control, apple postharvest disease, volatile organic compounds, antioxidant and defense-related enzymes, lactic acid bacteria

## Abstract

Apple black rot, a destructive postharvest disease caused by *Alternaria alternata*, poses significant economic threats during fruit storage and transportation. However, effective biocontrol bacteria to manage this disease remain limited. In this study, *Leuconostoc mesenteroides* strain GY-2, isolated from healthy apple fruit surfaces, had a remarkable biocontrol ability on apple black rot. While GY-2 exhibited no direct inhibitory effects in confrontation assays, volatile organic compounds (VOCs) emitted by the strain suppressed colony diameter of *A. alternata* by 70.8% in dual plate assays, indicating potent fungistatic activity. Notably, these VOCs produced by *L. mesenteroides* displayed broad-spectrum antifungal properties against multiple apple fungal pathogens. Microscopic analysis revealed that VOC exposure induced structural anomalies in *A. alternata* hyphae, including surface perforations and protoplast leakage, suggesting membrane integrity disruption. The VOCs produced by strain GY-2 were identified; four compounds had antifungal activities, among them, isoamylol exhibited the highest antifungal activity. Applying bacterial suspensions of strain GY-2 on apple fruit significantly reduced 91.4% of lesion areas of black rot. The strain exhibited robust colonization capacity on fruit surfaces, maintaining viable populations for over 15 days post-application, guaranteeing a sustained disease prevention. Furthermore, GY-2 treatment enhanced systemic resistance in apple fruit, as evidenced by upregulated antioxidant enzymes and defense-related enzymes. Importantly, application of GY-2 did not adversely affect key parameters of fruit quality, including firmness, soluble solids content, or acidity. These findings showed that the bacterial *L. mesenteroides* GY-2 was a promising biocontrol agent for managing postharvest black rot of apple fruit.

## 1. Introduction

Apples (*Malus domestica* Borkh.), renowned for their palatability, nutritional richness, and commercial significance, rank among the most globally consumed fruits [1]. However, prolonged storage and mechanical injuries during handling compromise fruit resistance, exacerbating postharvest decay and resulting in substantial economic losses [2,3]. Over 90 fungal species have been implicated in postharvest apple infections, with *Alternaria alternata*-induced black rot emerging as a particularly pervasive threat across major apple-producing regions [4,5,6,7]. Beyond direct fruit damage, *A. alternata* contamination raises critical food safety concerns, as the pathogen synthesizes hazardous mycotoxins, including alternariol monomethyl ether, alternariol, and tenuazonic acid, within infected tissues, posing risks to human health [8,9].

Previous research has focused on chemical interventions to manage *Alternaria*-induced apple rot. Ursolic acid showed significant antifungal efficacy by disrupting intracellular reactive oxygen species (ROS) homeostasis, compromising hyphal integrity, and altering membrane permeability [10]. Further investigations by Shu et al. (2021) revealed that epsilon-poly-L-lysine (ε-PL), a naturally derived cationic antimicrobial peptide, exhibited dose-dependent inhibition of *A. alternata* [11]. In parallel, β-aminobutyric acid (BABA) was identified as a resistance-inducing agent, effectively suppressing *A. alternata* proliferation through host defense activation [12]. Wang et al. (2023) highlighted the potential of tannic acid, a polyphenolic compound, which reduced lesion area by 84.2% at 5.0 mg mL^−1^ compared to untreated controls, underscoring its utility in postharvest black rot management [7].

Biological control represents an eco-sustainable strategy for managing postharvest diseases in perishable crops. Beneficial microorganisms, particularly lactic acid bacteria (LAB), have emerged as viable alternatives to synthetic fungicides due to their non-residual nature, biosafety, and environmental compatibility [13,14]. Historically employed in food fermentation, LAB exhibit broad-spectrum antimicrobial activity against spoilage microorganisms and phytopathogens [15]. Recognized as generally regarded as safe (GRAS) by the U.S. Food and Drug Administration and approved by the European Food Safety Authority, LAB holds food-grade safety credentials [16,17]. Their antagonistic effects are mediated through diverse bioactive metabolites, including bacteriocins, organic acids (e.g., phenylacetic acid), hydrogen peroxide, cyclic dipeptides, and short-chain fatty acids [18,19,20]. Bacteriocins, in particular, disrupt microbial viability by impeding nucleic acid/protein synthesis, compromising cell wall integrity, and enhancing membrane permeability [21,22]. Within the LAB family, *L. mesenteroides* has demonstrated significant biocontrol potential. For instance, *L. mesenteroides* subsp. *Mesenteroides* LB7 suppressed *Penicillium expansum* growth in vitro and reduced patulin contamination in apple juice [23]. Similarly, *L. mesenteroides* SNP037 mitigated soft rot in green peppers by antagonizing *Pectobacterium carotovorum* through bacteriocin production [24]. These findings underscore the versatility of *L. mesenteroides* strains in combating postharvest pathogens across diverse agricultural systems.

While lactic acid bacteria (LAB) have shown promise in managing postharvest diseases across various fruits and vegetables [25], their application against *Alternaria alternata*-induced apple black rot remains underexplored. This study aimed to (1) assess the in vitro antifungal activity of strain GY-2 against *A. alternata*, (2) identify the VOCs from strain GY-2 and test antifungal activities, (3) evaluate its control efficacy against black rot in postharvest apples, and (4) elucidate the underlying biocontrol mechanisms. Our findings not only advance the understanding of *L. mesenteroides*-mediated pathogen suppression but also lay the theoretical groundwork for deploying this strain as a sustainable solution in apple postharvest management, storage, and transportation.

## 2. Materials and Methods

### 2.1. Fruit and Pathogens

Mature ‘Fuji’ apples of uniform size and coloration, free from visible blemishes, were procured from a commercial orchard (38°49′21″ N, 115°26′31″ E) in Baoding, China. Selected fruit underwent surface sterilization via immersion in 2% (*v*/*v*) sodium hypochlorite solution for 10 min, followed by triple rinsing with sterile distilled water and aseptic air-drying on a laminar flow bench. The fungal pathogens, including *Alternaria alternata*, *Botryosphaeria dothidea*, *Trichothecium roseum*, *Valsa mali*, and *Colletotrichum fructicola*, were routinely maintained on potato dextrose agar (PDA) in the dark at 25 °C. For harvesting conidia, *A. alternata* colonies were gently abraded with a sterile loop under 10 mL sterile distilled water. The resulting suspension was filtered through sterile Miracloth (Calbiochem, Germany) and calibrated the spore density to 1 × 10^6^ conidia mL^−1^ using a hemocytometer.

### 2.2. Isolation Candidate Biocontrol Strains

Biocontrol strain isolation was performed from fresh apple fruit. The apples were sectioned into 1 × 0.5 × 0.5 cm^3^ fragments using a sterile scalpel. Five grams of tissue were homogenized with 10 mL of sterile distilled water in a mortar. Two milliliters of the resultant suspension were inoculated into 50 mL Luria–Bertani (LB) broth (Solarbio, Beijing, China) and incubated at 25 °C with 150 rpm for 48 h. Serial dilutions (10^−1^–10^−6^) of the enriched culture were prepared in sterile saline solutions, and 50 µL aliquots from each dilution were spread-plated onto PDA. Following 3–4 days of incubation at 25 °C, distinct colonies were purified via quadrant streaking on fresh PDA plates. A total of 45 pure isolates were cryopreserved in 20% (*v*/*v*) glycerol in a −80 °C refrigerator for long-term storage.

### 2.3. Assessment of the Inhibitory Activity of Strain GY-2 Against Apple Pathogens In Vitro

The antifungal spectrum of strain GY-2 was evaluated using the confrontation assay [26]. A 5-mm-diameter mycelial plug of *A. alternata*, *B. dothidea*, *T. roseum*, *V. mali*, and *C. fructicola* was centrally inoculated on PDA plates. Sterile filter paper discs (5 mm diameter) loaded with 5 μL of GY-2 suspension (10^8^ CFU mL^−1^) were positioned 3 cm radially from the fungal inoculum. Plates containing filter paper without bacterial suspension served as negative controls. All plates were incubated at 25 °C for 6 days, after which inhibition zones were quantified by measuring the radial distance between the fungal colony edge and the bacterial disc. Assays were performed in triplicate across three independent experimental runs.

The volatile-mediated antifungal activity of strain GY-2 was assessed using the dual plate assay [27]. The 100 μL of GY-2 suspension (10^8^ CFU mL^−1^) was spread-plated onto De Man, Rogosa, and Sharpe agar (MRS) plate. A parallel plate containing 100 μL sterile LB broth served as the negative control. The 5-mm in diameter pathogen disc was inoculated in the center of another MRS plate. Two plates were hermetically sealed with parafilm to allow volatile exchange while preventing physical contact, followed by incubation at 25 °C for 4–6 days. Fungal growth inhibition was quantified by measuring radial colony expansion relative to controls. Assays included triplicate biological replicates across three independent experimental runs.

### 2.4. Scanning Electron Microscopy

To elucidate the morphological alterations induced by volatile organic compounds (VOCs) on *A. alternata* hyphae, comparative scanning electron microscopy (SEM) was conducted on untreated and VOC-exposed specimens. Hyphal segments (5 × 5 mm^2^) from both treatment groups were excised using a sterile scalpel and fixed in 4% (*v*/*v*) glutaraldehyde (in 0.1 M phosphate buffer, pH 7.2) for 24 h at 4 °C. Following three rinses in phosphate-buffered saline (PBS, pH 7.4), samples underwent sequential dehydration in an ethanol gradient, with 15-min immersion at each concentration. Critical point drying was performed using liquid CO_2_ (Leica EM CPD300, Wetzlar, Germany) to preserve ultrastructural integrity. Dried specimens were mounted on aluminum stubs with conductive carbon tape, sputter-coated with 10 nm gold–palladium (Quorum Q150T ES, Ashford, UK), and imaged under high vacuum at 5 kV accelerating voltage using a field-emission SEM (Hitachi SU8100, Tokyo, Japan). A minimum of five biological replicates per treatment group were analyzed.

### 2.5. Identification of the Candidate Strain GY-2

The colonial morphology of strain GY-2 was characterized on LB agar plates following 48 h incubation at 25 °C. For ultrastructural analysis, bacterial cells were fixed in 2.5% (*v*/*v*) glutaraldehyde, dehydrated through an ethanol gradient, and sputter-coated with gold-palladium prior to imaging via field-emission SEM (Hitachi SU8100, Tokyo, Japan) at 5 kV accelerating voltage. Gram staining was performed using a standardized protocol [28]: cells were heat-fixed, stained with crystal violet (Solarbio, Beijing, China) for 1 min, treated with Gram’s iodine mordant (Solarbio, Beijing, China) for 1 min, decolorized with ethanol–acetone (Solarbio, Beijing, China) (3 s), and counterstained with safranin (Solarbio, Beijing, China) (30 s). Molecular identification involved genomic DNA extraction using the TIANamp Bacterial DNA Kit (Tiangen Biotech, Beijing, China). The 16S rDNA gene was amplified via PCR with universal primers 27F (5′-AGAGTTTGATCMTGGCTCAG-3′) and 1492R (5′-TACGGYTACCTTGTTACGACTT-3′) under the following conditions: initial denaturation at 95 °C (5 min); 35 cycles of 95 °C (30 s), 55 °C (30 s), 72 °C (90 s); final extension at 72 °C (10 min) [29]. Amplicons were sequenced bidirectionally (Sangon Biotech, Shanghai, China) and assembled using SeqMan v7.1.0. Phylogenetic construction was performed in MEGA11 via the maximum-likelihood algorithm with 1000 bootstrap replicates, incorporating strains from the NCBI GenBank database [30].

### 2.6. Analysis of Organic Compounds by Solid Phase Microextraction–Gas Chromatography–Mass Spectrometer (SPME-GC–MS)

The strain GY-2 was cultured in LB broth (Solarbio, Beijing, China) at 25 °C with 150 rpm for 48 h. The 20 μL bacterial suspensions of GY-2 were inoculated into a 10-mL headspace-vial containing 3 mL solid LB agar medium. The vial was added 20 μL of sterilized LB broth to the bottle as a control treatment. Each treatment had three repeats. The vials were kept at 25 °C for 5 days. Volatiles of each sample were extracted at 50 °C for 30 min using SPME fibers and characterized by SPME-GC–MS (ThermoFisher Scientific, Waltham, MA, USA) [31].

### 2.7. Analysis of Inhibitory Effect of Compounds on Mycelial Growth of A. alternata

Based on the detection results of GY-2 volatiles, we selected four compounds including Isopropanol, Isoamylol, Fenchone, Nerolidol purchased from Macklin Biochemical Co., Ltd. (Shanghai, China) to evaluate their antifungal activities against *A. alternata*. A 5-mm mycelial plug was obtained from the edge of an *A. alternata* colony using a punch and inoculated onto the center of PDA medium. Sterile filter papers containing different concentrations of the compounds were placed on the inner side of the Petri dish lids. The dishes were sealed with parafilm and incubated upside down at 28 °C. The tested concentrations of the compounds were set at 10, 20, 40, 60, and 80 μL per plate. A negative control without any compound was included. The colony diameters were observed and measured daily.

### 2.8. Effect of Strain GY-2 on Apple Black Rot

#### 2.8.1. Wound Inoculations

Surface-sterilized ‘Fuji’ apples were used to evaluate the biocontrol efficacy of strain GY-2 against black rot caused by *A. alternata*. Uniform wounds (3 × 3 × 3 mm^3^) were created at the equatorial region of each fruit using a white pipette tip (1 mm in diameter). A 15 μL aliquot of GY-2 suspension (1 × 10^8^ CFU mL^−1^) was pipetted into each wound using a micropipette, followed by inoculation with 15 μL *A. alternata* conidial suspension (1 × 10^6^ conidia mL^−1^) after 2 h of air-drying, and this group of treatment was represented by Aa+GY-2. The fruit only inoculated with conidial suspensions of *A. alternata* was used as a positive control, and this group of treatment was represented as Aa; the fruit inoculated with GY-2 bacterial suspensions was represented as GY-2. The fruit inoculated with equivalent volumes of sterile LB broth (LB) was used as a negative control. All fruits were placed in humidity-controlled plastic boxes (90 ± 5% RH) lined with water-saturated filter paper and incubated at 25 °C. Disease progression was quantified at 7 days post-inoculation (DPI) by measuring lesion diameter (mm) using digital calipers and calculating necrotic area (mm^2^). The experiment comprised fifteen fruit per treatment across three independent trials.

#### 2.8.2. Inoculations Without Wounds

To simulate natural infections, intact surface-sterilized ‘Fuji’ apples were employed to assess the preventive efficacy of strain GY-2 against *A. alternata*-mediated black rot. Test fruit were uniformly sprayed with GY-2 suspensions (1 × 10^8^ CFU mL^−1^) using a sterile spray bottle, ensuring complete surface coverage. Control groups received equivalent applications of sterile distilled water. Following a 2-h air-drying period under laminar flow, fruit were inoculated via fine mist spraying with *A. alternata* conidial suspension (1 × 10^6^ conidia mL^−1^). All fruit were maintained in humidity-regulated chambers (90 ± 5% RH) at 25 °C for 15 days. Disease parameters, including disease incidence (infected fruit/total fruit), lesion number, and necrotic area, were quantified at 15 DPI. The experimental design comprised three independent trials, each containing 30 apple fruit per treatment group.

### 2.9. Colonization of Strain GY-2 on Apple Fruit

The colonization capacity of strain GY-2 on apple tissues was evaluated using the dilution-plate method [32]. For wound colonization analysis, a dose of 15 μL of GY-2 suspension (1 × 10^8^ CFU mL^−1^) was applied to artificial wounds on apple surfaces. To assess peel colonization, intact apples were immersed in the bacterial suspension for 5 min. Sterile water-treated apples served as negative controls. All inoculated apples were incubated at 25 °C. Apple tissues (1 g aliquots from either wounded areas or peels) were collected at 0, 1, 2, 4, 8, and 16 DPI. Samples were homogenized in 5 mL of sterile water using a grinder. Subsequently, serial 10-fold dilutions were prepared, and 0.1 mL aliquots from each dilution were spread onto LB agar plates. After 48-h incubation at 25 °C, viable bacterial colonies were enumerated. Colony counts were normalized to log_10_ CFU per gram fresh weight. The experimental design included three technical replicates per time point, with the entire experiment independently repeated three times.

### 2.10. Effect of Strain GY-2 on Defense-Related Enzyme Activities

To investigate host physiological responses, 15 μL aliquots of either GY-2 bacterial suspension (1 × 10^8^ CFU mL^−1^) or sterile water (negative control) were inoculated into artificial wounds on apple fruit. Tissue samples (0.1 g) surrounding the inoculation sites were collected at designated time intervals. The excised tissues were cryogenically homogenized using liquid nitrogen prior to enzymatic analysis. The activities of key defense-related enzymes, including superoxide dismutase (SOD), peroxidase (POD), catalase (CAT), polyphenol oxidase (PPO), and phenylalanine ammonia-lyase (PAL), were quantitatively determined using commercial assay kits of SOD activity, POD activity, CAT activity, PPO activity, PAL activity (Solarbio Life Science, Beijing, China) following manufacturer protocols [26].

### 2.11. Effect of Strain GY-2 on the Postharvest Apple Quality

To assess the effect of strain GY-2 on ‘Fuji’ apple quality, surface-sterilized fruit were subjected to 1-min immersion in GY-2 suspension (1 × 10^8^ CFU mL^−1^). Fruit treated with sterile water under identical conditions served as controls. All samples were subsequently maintained under controlled environmental conditions (25 °C, 90% RH) for quality parameter analysis.

Key postharvest indices, including titratable acidity (TA), total soluble solids (TSS), flesh firmness, and weight loss percentage, were measured at 15 DPI. These determinations followed established protocols [26], employing standardized methodologies: TA quantification via acid-base titration, TSS assessment using digital refractometry, firmness evaluation through penetrometer analysis, and weight loss calculation based on initial and final mass measurements. The experiment was repeated three times, and each treatment had three apples.

### 2.12. Statistical Analysis

Data processing and statistical analyses were implemented by employing IBM SPSS Statistics software (v26.0, IBM, New York, NY, USA). Between-group comparisons were conducted through independent samples *t*-tests to assess mean differences between experimental and control groups, with statistical significance set at *p* < 0.05. For multi-group comparisons, one-way ANOVA with least significant difference (LSD) testing was implemented, maintaining the same significance threshold (*p* < 0.05) for all pairwise comparisons.

## 3. Result

### 3.1. Evaluation of Biocontrol Effects of Strain GY-2 In Vitro

**Figure 1:**
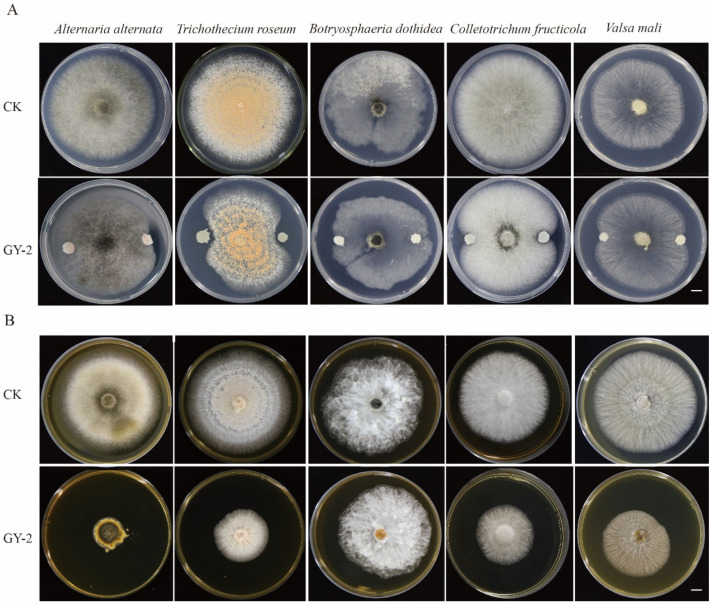
Antagonistic effects of strain GY-2 and its volatile organic compounds on the hyphae growth of apple pathogens. (**A**) The confrontation assay of strain GY-2 against pathogens in PDA plates. (**B**) Assay of the inhibitory effect of VOCs from strain GY-2 against the same set of apple pathogens using dual plate assay. The types of different apple pathogens are listed at the top of (**A**,**B**). Scale bars (**A**,**B**) = 5 mm.

**Figure 2:**
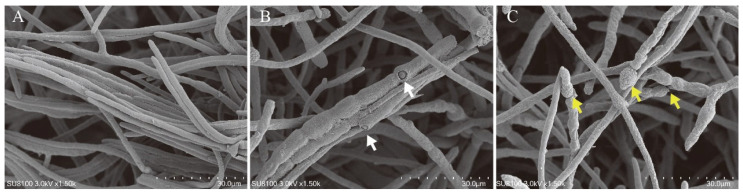
Ultrastructural alterations in *A. alternata* hyphae induced by VOCs from strain GY-2. (**A**) Untreated control hyphae exhibiting typical morphology: slender filaments with smooth surfaces. (**B**,**C**) VOC-treated hyphae demonstrating structural deformities, including abnormal thickening, surface pitting (white arrows), and protoplast leakage (yellow arrows). Scale bars: 30 μm (**A**–**C**).

A total of 91 microbial strains were isolated from apple tissues. The confrontation assay and dual plate assay were used to screen candidate biocontrol strains. Among the tested strains, a strain GY-2 showed no antagonistic activities towards hyphae of *A. alternata*, *B. dothidea*, and little inhibitory activities against *T. roseum*, *C. fructicola*, and *V. mali* in confrontation assay (Figure 1A; Table 1); however, its released VOCs in dual plate assay had antifungal activities against hyphae of five apple pathogens in different degrees; strain GY-2 had the strongest inhibitory effect on *A. alternata*, and its inhibition rate reached 70.8% (Figure 1B; Table 1). The edge of normal hyphae and inhibited hyphae of *A. alternata* in dual plate assay were further observed by an SEM, the normal hyphae were slender, its hyphal surface was smooth (Figure 2A), while the hyphae inhibited by VOCs became thicker and rough, there were some holes on the hyphal surface, and the hyphae had protoplast leakages (Figure 2B,C), indicating that VOCs from GY-2 can inhibit hyphal growth of *A. alternata*, break the hyphal structure and cause protoplast leakage.

### 3.2. Identification of Strain GY-2

**Figure 3:**
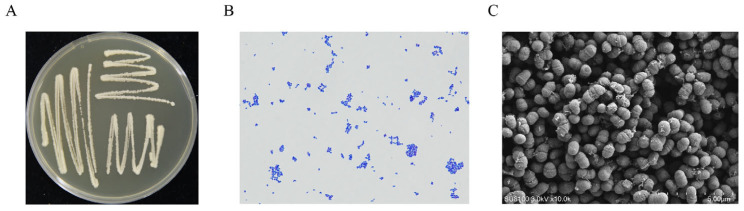
Morphological identification and phylogenetic analysis of strain GY-2. (**A**) Colonial morphology of GY-2 on LB agar after 48 h incubation at 28 °C. (**B**) Gram staining of strain GY-2. (**C**) Bacterial morphology of strain GY-2 by SEM examination (bar = 5 μm).

**Figure 4:**
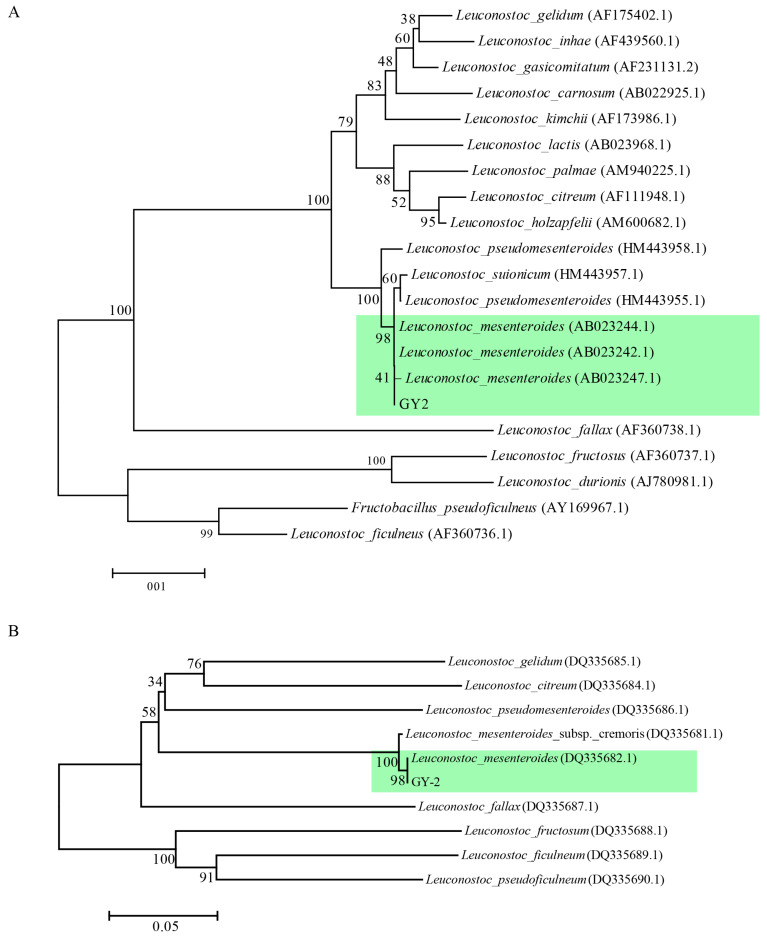
Phylogenetic analysis of strain GY-2. (**A**) A phylogenetic tree based on 16S rDNA sequence. (**B**) A phylogenetic tree based on gyrB sequence. The green colors indicate taxonomic status of GY-2.

Strain GY-2 exhibited distinct colonial morphology on LB agar plates, forming opaque ivory-white colonies with regular margins after 48 h incubation at 28 °C (Figure 3A). Gram-staining characterization identified it as a Gram-positive bacterium (Figure 3B). SEM revealed ovoid-shaped cellular morphology, with dimensional measurements ranging from 0.7 to 1.2 μm (length) × 0.5 to 0.7 μm (width) (Figure 3C). Phylogenetic reconstructions based on the 16S rDNA gene and gyrB gene sequencing positioned the strain within the *L. mesenteroides* evolutionary clade (Figure 4A,B). Combined phenotypic and genotypic evidence conclusively identified strain GY-2 as *L. mesenteroides*.

### 3.3. Identification of VOCs Emitted from GY-2 and Its Antifungal Activity

**Figure 5:**
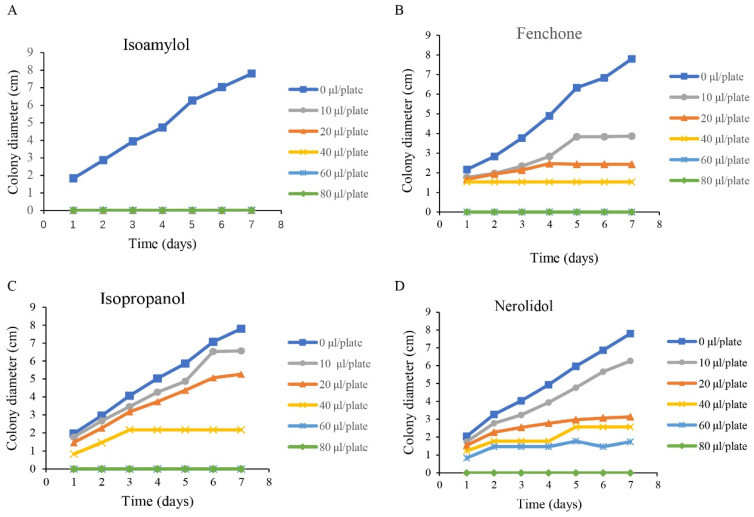
The effects of different compounds on the hyphal growth of *A. alternata*. (**A**) Isoamylol; (**B**) Fenchone; (**C**) Isopropanol; (**D**) Nerolidol.

The components of VOCs released from GY-2 were analyzed by SPME combined with GC-MS. A total of four compounds were identified, including isopropanol, isoamylol, fenchone, and nerolidol (Table 2). Four compounds were tested for their in vitro antifungal activity against *A. alternata*. All the compounds had antifungal activities. Among them, isoamylol had the strongest antifungal activity (Figure 5), and it could completely inhibit the hyphal growth of *A. alternata* at less than 10 μL/plate.

### 3.4. Biocontrol Activity of L. mesenteroides GY-2 Against A. alternata in Apple Fruit

**Figure 6:**
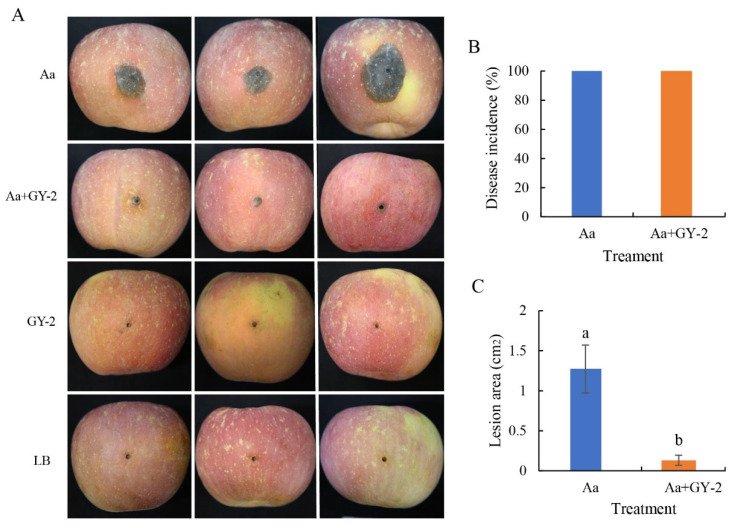
Effect of *L. mesenteroides* GY-2 on the occurrence of apple black rot. (**A**) Photographs of diseased apple fruit were taken 7 days after treatments. Aa indicates the fruit were inoculated with *Alternaria alternata* (Aa); Aa+GY-2 indicates the fruit were inoculated with *A. alternata* and GY-2 bacterial suspensions; GY-2 indicates the fruit were inoculated with GY-2 bacterial suspensions; LB indicates the fruit were inoculated with LB broth (Negative control). (**B**) Disease incidence of black rot after inoculation of Aa and Aa+GY-2 at 7 DPI, respectively. (**C**) Lesion areas (**C**) of black rot with the same treatments at 7 DPI. (a, b) indicates significant differences at *p* < 0.05.

To evaluate the effect of strain GY-2 on black rot caused by *A. alternata*, the strain GY-2 cell suspensions were applied to the wounds of apple fruit before the application of conidial suspensions of *A. alternata*. At 7 DPI, the fruit only inoculated with *A. alternata* (positive control group) developed an obvious brown lesion around the inoculation sites, whereas the apple fruit treated with strain GY-2 before *A.alternata* application formed a tiny round lesion with a diameter of 1–3 mm (Figure 6A). Both the positive control group and the GY-2-treated group showed black rot (Figure 6B). However, the lesion area in the GY-2 group was reduced by 91.4% in comparison with the positive control group at 7 DPI (Figure 6C), suggesting that strain GY-2 bacterial suspensions can effectively inhibit the expansion of black rot. The apple tissues around the inoculation sites by the GY-2 bacterial treatment had no symptoms, which was similar to LB treatment, indicating that GY-2 application did not have harmful effects on apple fruit (Figure 6A).

To further elucidate the protective efficacy of strain GY-2 under natural infection conditions, we sprayed the bacterial suspensions of GY-2 on intact apple fruit. At 15 DPI, co-application of GY-2 with *A. alternata* exhibited a 36.6% reduction in disease incidence compared to the pathogen-only control (Table 3). Notably, quantitative analyses revealed that the lesion numbers in the GY-2 and *A. alternata* group decreased 30.8% compared with the positive control group (Table 3). The total of lesion areas of the GY-2 and *A. alternata* group reduced by 60.3% compared with the *A. alternata*-treated group (Table 3). These findings collectively indicate that application of GY-2 bacterial suspension effectively mitigates black rot progression under natural infection scenarios.

### 3.5. Colonization of L. mesenteroides GY-2 on Apple Fruit

**Figure 7:**
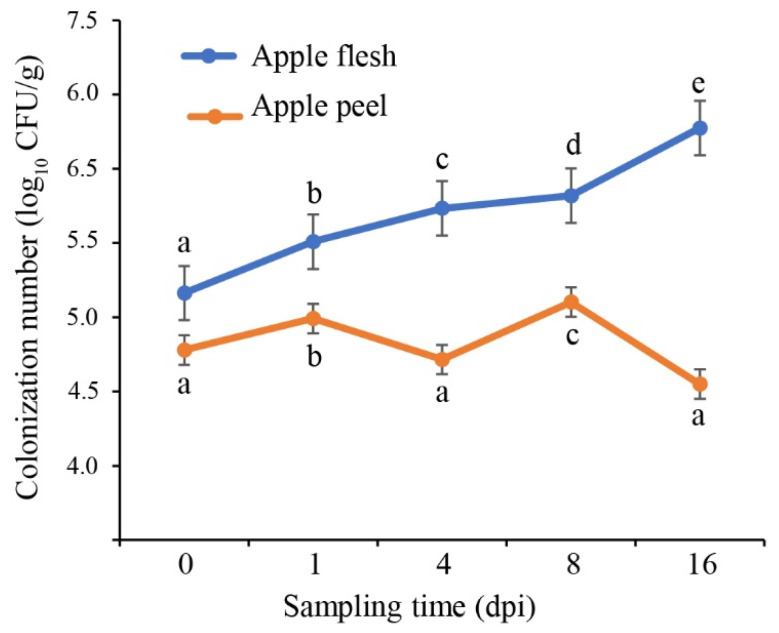
Population dynamics of *L. mesenteroides* GY-2 in flesh and peels of apple fruit during sampling time at 0, 1, 4, 8, and 16 DPI. Different lowercase letters indicate statistically significant differences at *p* < 0.05.

To investigate the colonization dynamics and population kinetics of strain GY-2 in apple tissues, we quantified its growth patterns in both flesh and peel. In flesh tissues, GY-2 exhibited progressive proliferation, with its population density increasing by 12.8-fold from 0 to 16 DPI (Figure 7). In contrast, peel colonization showed initial bacterial counts stabilized at 1.9 × 10^5^ CFU g^−1^ at 0 DPI, followed by a moderate reduction to 1.1 × 10^5^ CFU g^−1^ by 16 DPI (Figure 7). These quantitative results demonstrate strain GY-2’s effective colonization capacity in both internal and external apple fruit tissues, with notably stronger proliferation observed in flesh compared to peels.

### 3.6. Effect of L. mesenteroides GY-2 on Antioxidant Enzymes and Defense-Related Enzymes in Apple

**Figure 8:**
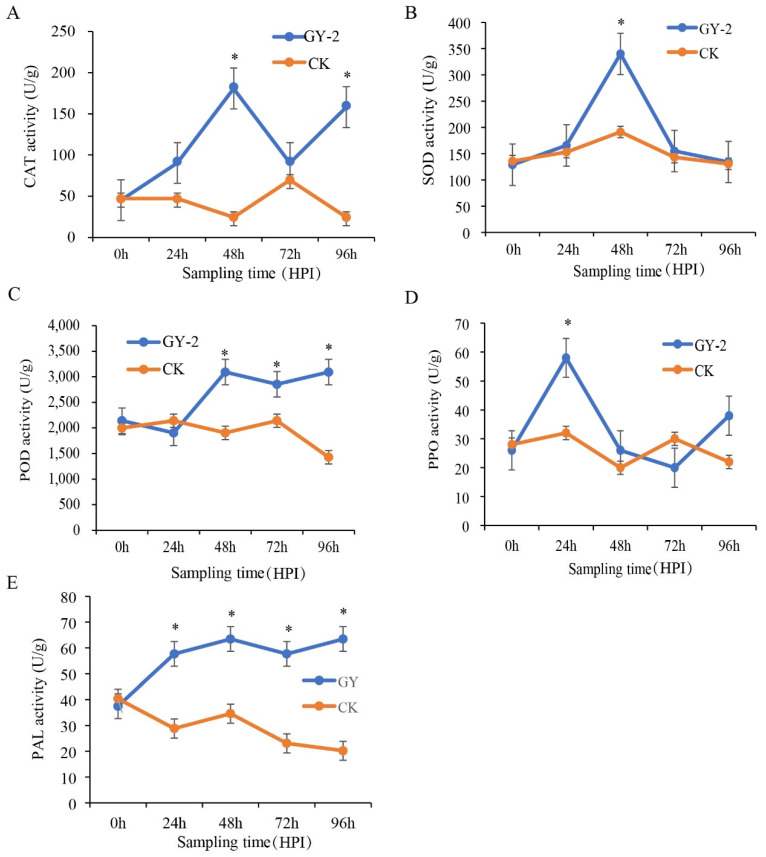
Catalase (CAT, (**A**)), superoxide dismutase (SOD, (**B**)), peroxidase (POD, (**C**)), polyphenol oxidase (PPO, (**D**)), and phenylalanine ammonia-lyase (PAL, (**E**)) activities in GY-2 strain-treated apple fruit from 0 to 96 HPI. GY-2: apple fruit pre-treated with only *L. mesenteroides* GY-2. Water: apple fruit pre-treated with only water. Asterisk (*) indicates significant differences at *p* < 0.05.

To evaluate the biocontrol potential of *L. mesenteroides* GY-2 through modulation of host enzymatic defenses, we systematically quantified three key antioxidant enzymes (CAT, SOD, POD) and two defense-related enzymes (PPO, PAL) in apple fruit tissues. CAT activity in GY-2-inoculated apples showed marked enhancement, reaching levels 8.0-fold and 7.0-fold higher than sterile water controls at 48 and 96 h post-inoculation (HPI), respectively (Figure 8A). Similarly, SOD activity increased 1.8-fold relative to controls at 48 HPI (Figure 8B). Peroxidase (POD) activity exhibited sustained upregulation, with 9.0-fold and 8.4-fold elevations observed at 48 and 96 HPI (Figure 8C). PPO activity in GY-2-treated fruit surpassed control levels as early as 24 HPI (Figure 8D). Notably, PAL demonstrated progressive activation, maintaining significantly higher activity than controls across all monitored timepoints (24–96 HPI) (Figure 8E). These coordinated enzymatic inductions collectively demonstrate GY-2’s capacity to amplify both antioxidant defense systems and pathogenesis-related pathways in apple fruit.

### 3.7. Effects of L. mesenteroides GY-2 on Fruit Quality

**Figure 9:**
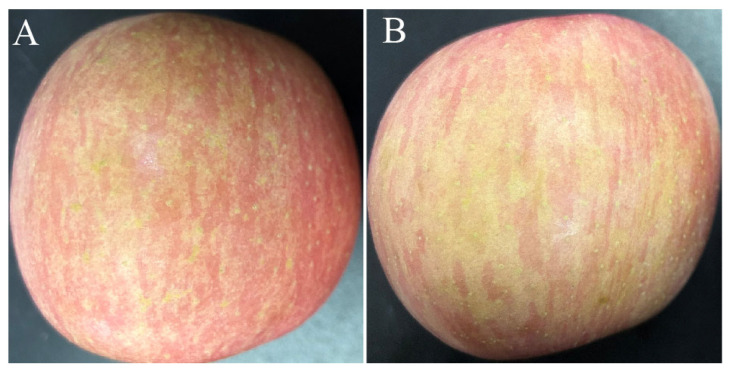
Effects of *L. mesenteroides* GY-2 on fruit quality. (**A**) The fruit treated with GY-2 bacterial suspension. (**B**) The fruit treated with sterile water.

As shown in Table 3, there were no obvious differences in acidity, soluble solid content, firmness, and the weight loss rate between apple fruit treated with strain GY-2 and those from the CK group. (Figure 9; Table 4). These findings suggested that strain GY-2 had no discernible impact on the apple postharvest quality.

## 4. Discussion

Apple fruit are susceptible to *A. alternata* infections during the postharvest phase [5]. With the increasing demand for food safety and eco-friendliness, biocontrol agents have emerged as an ideal strategy for managing postharvest black rot. Previous studies have identified promising microbial candidates for controlling this disease. Tulukoğlu-Kunt et al. (2023) isolated thirteen epiphytic yeast strains of *Aureobasidium pullulans* from apple fruit, and these isolates significantly restrained lesion development of black rot, some strains achieving 100% lesion reduction rates [33]. Bektas et al. (2024) screened twenty-six endophytic *Bacillus* sp. strains from apple fruit, reporting that co-inoculation of *B. myloliquefaciens* strain IB1 and *B. licheniformis* strain IB21 with the pathogen resulted in 81.8% disease suppression [34]. In this study, we expanded the repertoire of potential biocontrol agents by isolating indigenous microorganisms from apple fruit. A candidate strain designated GY-2, identified as *L. mesenteroides* through comprehensive morphological characterization, Gram staining, and 16S rDNA phylogenetic analysis, demonstrated exceptional biocontrol performance against black rot. Notably, strain GY-2 achieved a control efficacy exceeding 90% under experimental conditions. To our knowledge, this represents the first documented application of *L. mesenteroides* for the suppression of *A. alternata* infections. These findings enrich the diversity of microbial resources available for postharvest disease management in apples, providing a novel lactic acid bacterium-based alternative to conventional control methods.

Previous studies reported that *L. mesenteroides* strains could directly inhibit fungal and bacterial pathogens of fruit and vegetables, such as *Penicillium expansum*, *Listeria monocytogenes*, *Allorhizobium vitis*, *Erwinia amylovora*, and *Pectobacterium carotovorum* [23,24,35,36]. Our findings are not consistent with previous studies. It is found that *L. mesenteroides* strain GY-2 did not inhibit the hyphal growth of *A. alternata*, indicating that the mechanism for suppression of apple black rot was not antifungal effects. Many studies indicated that antagonistic microorganisms can suppress the growth of pathogens after fruit and vegetable harvesting by generating volatile organic compounds (VOCs) that inhibit conidial germination and hyphal expansion, disrupt cell structures, and interfere with normal metabolism [37,38]. In this study, VOCs released from the GY-2 colony significantly inhibited the hyphal growth of *A. alternata* in vitro. Our findings showed that all the compounds, including isopropanol, isoamylol, fenchone, and nerolidol, released from strain GY-2 had antifungal activities. The hyphae of *A. alternata* became thicker and rougher wth VOC treatment, and VOCs caused holes and protoplast leakage of hyphae. These results suggested that one of the mechanisms for controlling apple black rot was the diffused antifungal VOCs from *L. mesenteroides* strain GY-2. To our knowledge, this is the first time to report VOCs from *L. mesenteroides* having antifungal activities. In addition, previous studies suggested that the VOCs emitted by microorganisms are different depending on the medium and the organisms with which they interact [39]. In this study, we only identified the volatile compounds isolated by growing the GY2 bacterium in LB medium. We will characterize the VOCs of GY-2 during interaction with *A. Alternaria* in our future plan.

The colonization capacity of biocontrol bacteria serves as a critical mechanism for pathogen suppression through competitive exclusion of nutrients and spatial occupation on fruit surfaces [40,41]. In this study, strain GY-2 showed successful colonization in both wound sites and intact peel surfaces of apples. Notably, colonization density at wound sites significantly exceeded that on intact peels, likely attributable to the abundance of nutrient exudates at damaged fruit tissues. This spatial colonization pattern aligns with observations of *B. velezensis* JZ51 on apple fruit [26]. These findings imply that competition for nutrients and physical space between strain GY-2 and *A. alternata* at wound sites represents a key mechanism underlying disease suppression. Furthermore, strain GY-2 exhibited sustained colonization capacity, maintaining viable populations in apple wounds and on peel surfaces for over 15 days. This extended colonization capability highlights its potential for long-term prophylactic protection against *A. alternata* infection during postharvest storage, offering a durable biological barrier against pathogen establishment.

Antioxidant enzymes play a critical role in mitigating oxidative stress by scavenging reactive oxygen species (ROS) in postharvest fruit tissues [42,43]. Substantial evidence highlights the pivotal function of enzymes such as POD, CAT, and SOD in bolstering fruit disease resistance through protection against oxidative damage [44,45,46,47]. CAT, a central component of the plant antioxidant system, regulates intracellular ROS homeostasis by decomposing hydrogen peroxide (H_2_O_2_). Pathogen invasion triggers a surge in H_2_O_2_ production, inducing lipid peroxidation of cellular membranes and oxidative degradation of biomacromolecules. By efficiently scavenging H_2_O_2_, CAT attenuates oxidative injury and maintains cellular integrity. Furthermore, CAT participates in plant defense signaling cascades, where its activity modulates downstream resistance responses. For instance, CAT deficiency exacerbates disease symptoms and cell death in tobacco, while its overexpression enhances pathogen resistance [48]. This study revealed that treatment with *L. mesenteroides* strain GY-2 significantly upregulated CAT activity in apple fruit, indicating its capacity to potentiate host defense. Beyond antioxidant enzymes, pathogenesis-related enzymes also contribute critically to fruit resistance. PAL, the rate-limiting enzyme in phenylpropanoid biosynthesis, governs the production of antifungal metabolites linked to disease resistance, with its activity directly correlating with plant defense potency [43]. Similarly, polyphenol oxidase (PPO) enhances resistance by oxidizing phenolics into toxic quinones that inhibit fungal proliferation. Our findings demonstrate that strain GY-2 markedly induced both PAL and PPO activities, indicating that GY-2 can inhibit the infection of *A. alternata* by enhancing the activities of defense-related enzymes. Collectively, these results establish that strain GY-2 suppresses apple black rot not only through volatile-mediated antifungal effects but also via systemic induction of fruit resistance.

The commercial viability of biocontrol agents necessitates rigorous evaluation of their impacts on postharvest fruit quality, a critical determinant for agricultural applications [49,50]. While emerging studies report microbial efficacy against apple black rot [33,34], systematic assessments of quality preservation remain conspicuously absent from the current research paradigm. Our study addresses this knowledge gap through comprehensive quality metrics analysis, revealing that *L. mesenteroides* GY-2 treatment maintained key postharvest quality parameters in apples. Moreover, *L. mesenteroides* belongs to LAB, which holds food-grade safety credentials [16,17]. It has been widely utilized in the production of various fermented foods such as sauerkraut (pickled cabbage), kimchi (a traditional Korean food), and cheese, and is employed as a starter culture for kimchi fermentation [51,52,53]. Consequently, it is generally recognized as harmless to humans, animals, and the environment. These findings establish strain GY-2 as a biocontrol agent that concurrently suppresses pathogenicity of *A. alternata* and preserves commodity value of apple fruit.

## 5. Conclusions

This study presents the isolation and characterization of *L. mesenteroides* GY-2, a novel biocontrol agent derived from apple fruit tissues, demonstrating significant potential for managing *A. alternata*-induced apple black rot. Strain GY-2 exhibited long-term tissue colonization on apple fruit. GY-2-derived VOCs inhibited the growth of hyphae and altered the hyphal morphology of *A. alternata*. The antifungal VOCs of strain GY-2 were isopropanol, isoamylol, fenchone, and nerolidol. Furthermore, GY-2 treatment enhanced host defense capacity through the coordinated upregulation of antioxidant enzymes and defense-related enzymes. Importantly, the application of *L. mesenteroides* to apples did not affect fruit quality. These results position GY-2 as a multifaceted biocontrol candidate, combining VOC-mediated pathogen suppression with host resistance priming, all while maintaining fruit marketability. Our findings not only advance the understanding of *L. mesenteroides*-mediated pathogen suppression but also lay the theoretical groundwork for deploying this strain as a sustainable solution in apple postharvest management, storage, and transportation.

## Figures and Tables

**Table 1:** Inhibition rates of GY-2 culture blocks and VOCs against the hyphae growth of five apple pathogens.

Pathogens	Inhibition Rates of Confrontation Assay (%)	Inhibition Rates of VOCs (%)
*Alternaria alternata*	0	70.8
*Trichothecium roseum*	40.4	52.1
*Botryosphaeria dothidea*	0	11.1
*Colletotrichum fructicola*	29.8	39.1
*Valsa mali*	16.7	51.0

**Table 2:** Volatile organic compounds of strain GY-2 were identified by HS-SPME/GC-MS analysis.

Name	Retention Time	CAS No.	Molecular Formula	Molecular Weight
Isopropanol	2.08	67-63-0	C_3_H_8_O	60.1
Isoamylol	3.95	123-51-3	C_5_H_12_O	88.15
Fenchone	5.15	1195-79-5	C_10_H_16_O	152.23
Nerolidol	16.24	142-50-7	C_15_H_26_O	222.37

**Table 3:** Effect of *L. mesenteroides* strain GY-2 bacterial suspensions on black rot in naturally infected conditions at 15 DPI.

Treatment	Disease Incidence (%)	Lesion Number	Total of Lesion Area (mm^2^)
Aa	63.3	13	290.7
Aa+GY-2	26.7	9	115.3

**Table 4:** Effects of strain GY-2 on apple fruit quality.

Treatment	Acidity (%)	Soluble Solid Content (%)	Firmness (N)	Weight Loss Rate (%)
CK	0.90 ± 0.12	13.57 ± 1.69	6.58 ± 0.72	3.43 ± 1.29
GY-2	0.92 ± 0.02	13.87 ± 1.16	7.12 ± 1.06	4.13 ± 0.52

## Data Availability

The original contributions presented in this study are included in the article. Further inquiries can be directed to the corresponding author.
